# Chronic nigral neuromodulation aggravates behavioral deficits and synaptic changes in an α-synuclein based rat model for Parkinson’s disease

**DOI:** 10.1186/s40478-019-0814-3

**Published:** 2019-10-22

**Authors:** Teresa Torre-Muruzabal, Jens Devoght, Chris Van den Haute, Bert Brône, Anke Van der Perren, Veerle Baekelandt

**Affiliations:** 1 KU Leuven, Laboratory for Neurobiology and Gene Therapy, Department of Neurosciences, Leuven Brain Institute, Leuven, Belgium; 2 UHasselt, BIOMED, Hasselt, Belgium; 3 KU Leuven, Leuven Viral Vector Core, Leuven, Belgium

**Keywords:** α-Synuclein, DREADDs, Neuronal activity, Parkinson’s disease

## Abstract

Aggregation of alpha-synuclein (α-SYN) is the pathological hallmark of several diseases named synucleinopathies, including Parkinson’s disease (PD), which is the most common neurodegenerative motor disorder. Alpha-SYN has been linked to synaptic function both in physiological and pathological conditions. However, the exact link between neuronal activity, α-SYN toxicity and disease progression in PD is not clear. In this study, we aimed to investigate the effect of chronic neuromodulation in an α-SYN-based rat model for PD using chemogenetics. To do this, we expressed excitatory Designer Receptors Exclusively Activated by Designer Drugs (DREADDs) combined with mutant A53T α-SYN, using two different recombinant adeno-associated viral (rAAV) vectors (serotypes 2/7 and 2/8) in rat substantia nigra (SN) and investigated the effect on motor behavior, synapses and neuropathology. We found that chronic neuromodulation aggravates motor deficits induced by α-SYN, without altering dopaminergic neurodegeneration. In addition, neuronal activation led to changes in post-translational modification and subcellular localization of α-SYN, linking neuronal activity to the pathophysiological role of α-SYN in PD.

## Introduction

Alpha-synuclein (α-SYN) is considered to be a key player in Parkinson’s Disease (PD) and other neurodegenerative disorders like dementia with Lewy bodies (DLB) and multiple system atrophy (MSA) called together synucleinopathies [1–3]. Alpha-SYN deposits, called Lewy bodies (LB) in neurons or glial cytoplasmic inclusions (GCI) in oligodendrocytes are the main pathological hallmark of this group of diseases [1, 2, 4]. Since the discovery of α-SYN more than 30 years ago both the physiological and pathological role of this protein has been widely studied, nevertheless, many questions remain unanswered [1, 5, 6]. One important physiological function attributed to α-SYN is the chaperone function in the formation of the SNARE complex [7–10]. Supporting this hypothesis, mice lacking α-SYN have abnormal synaptic structure, transmission and age-dependent neuronal dysfunction [11]. Furthermore, overexpression of α-SYN has been shown to affect vesicle recycling [12, 13].

Alpha-SYN exists in different conformations. The precise conformation of α-SYN in physiological conditions has been heavily debated [14–16]. In pathological conditions, monomers aggregate into β-sheet-containing fibrillar forms probably via oligomeric intermediate species. Which of these species is most toxic for the cell is still argued [17–20]. Recently, different recombinant fibrillar forms of α-SYN or ‘strains’ displaying distinct phenotypic properties have been produced and extensively characterized in vitro as well as in vivo [3, 21]. This suggests that these distinct fibrillar forms of α-SYN could be involved in the development of different synucleinopathies, all characterized by the aggregation of one single protein, α-SYN [22, 23]. Next to the structural conformation of α-SYN, post-translational modifications such as phosphorylation of α-SYN at serine129 (P-S129 α-SYN) are pathological makers in PD. However, how this phosphorylation alters the aggregation and toxicity inducing properties of α-SYN, is still debated [24–26]. Extracellular transmission or spreading of α-SYN was suggested for the first time when the presence of LB pathology was observed in the brain of patients with embryonic cell transplantation [27, 28]. This cell-to-cell spreading supports Braak’s hypothesis, staging disease progression based on the topographical spreading of LB pathology throughout the brain [29–31]. These findings were further supported using cell culture and as well as in vivo models [32, 33].

Synaptic deficits in the dopaminergic synapses have also been linked to PD pathogenesis. On the one hand, there is ample evidence for a physiological role of multimeric α-SYN at the synapse, where it is enriched [7, 34]. On the other hand, fibrillar pathogenic forms of α-SYN seem to inhibit the formation of the SNARE complex, as opposed to physiological forms of α-SYN [35]. In PD patients, proteomic analysis of cerebrospinal fluids found a distinct expression pattern of synaptic proteins between PD patients and controls [36–38]. In α-SYN overexpression animal models, mainly changes in dopamine (DA) transmission are reported consisting of both a reduction in DA re-uptake and DA release [39, 40]. Furthermore, a reduction in the number of dopaminergic synapses as well as depletion of synaptic vesicles are described after α-SYN overexpression in the substantia nigra (SN) dopaminergic neurons (DN) [13]. Electrophysiological recordings indicate an increase in pacemaking activity specifically in SN DN after α-SYN overexpression [41]. Dopaminergic signaling in the striatum (STR) is key to proper motor control which is severely impaired in PD. DA denervation in the STR causes a disruption of the modulatory role of DA in the tripartite synapses formed between glutamatergic neurons and medium spiny neurons (MSN) [42, 43]. These non-DA cell types are shown to have a 30–50% decrease in dendritic spines and conversely, an increased activity in the corticostriatal system in parkinsonian models [44, 45]. However, how these changes in both dopaminergic as well as glutamatergic synapses affect α-SYN pathology, spreading and synaptic function remains unclear.

In this study, we aimed to investigate the link between neuronal activity and α-SYN pathology. Using a well-characterized rat model for PD, based on viral vector-mediated overexpression of α-SYN in the SN [46], we opted to chronically modulate neuronal activity and study the effect on α-SYN pathology, spreading and neurotoxicity. To modulate the neuronal activity in vivo, we used Designer Receptors Exclusively Activated by Designer Drugs (DREADDs) technology [47]. These are G-protein coupled receptors (GPCRs), which are selectively activated by an inert compound clozapine-N-oxide (CNO). Several types of DREADDs are currently available and differ in the GPCR cascades they activate [48]. This technique is now broadly used in the field of neuroscience. The non-invasive activation of neuronal populations and the longer lasting effect is of added value compared to optogenetics. In our study, we expressed the transgenes in our target population using adeno-associated viral vectors and made use of hM3Dq, which activates a Gq cascade resulting in increased neuronal activity. The hM3Dq is the most commonly used DREADD receptor to activate neuronal populations [49]. First, we thoroughly validated neuromodulation of nigral DN in the rat brain. Second, we applied this technology in our α-SYN-based PD model. We found that chronic neuronal activation aggravates α-SYN induced motor deficits in vivo. Further, we found that chronic neuronal activation increases soluble α-SYN levels in the STR and induces changes in synaptic proteins.

## Materials and methods

### Recombinant AAV production and purification

Vector production and purification was performed as previously described [50]. For the α-SYN and GFP vectors, the plasmids include: the constructs for the rAAV2/7 serotype, the AAV transfer plasmid encoding the human A53T mutant α-SYN or the enhanced green fluorescent protein (eGFP) under the control of the CMVie-enhanced synapsin1 promoter and the pAdvDeltaF6 adenoviral helper plasmid. For the DREADD vector, plasmids include: the constructs for the AAV2/8 serotype, the hM3Dq-mCherry (hM3Dq plasmid was based on pAAV-hSyn-DIO-hM3D(Gq)-mCherry, gift from Bryan Roth (Addgene plasmid # 44361; http://n2t.net/addgene:44361; RRID:Addgene_44,361)) [47] or mCherry transgene under control of the mouse CamKII promoter as well as the pAdvDeltaF6 adenoviral helper plasmid. Real-time polymerase chain reaction analysis was used for genome copy (GC) determination.

### rAAV vector injection and CNO treatment

All animal experiments were carried out in accordance with the European Communities Council Directive of November 24, 1986 (86/609/EEC) and approved by the Bioethical Committee of the KU Leuven (Belgium) (ECD project 2017–070). Young adult female Wistar rats (Janvier, France) weighing about 200 to 250 g were housed under a normal 12-h light/dark cycle with free access to pelleted food and tap water. All surgical procedures were performed using aseptic techniques and ketamine (60 mg/kg intraperitoneal [i.p.], Ketalar, Pfizer, Belgium) and medetomidine (0.4 mg/kg, Dormitor, Pfizer) anesthesia. Following anesthesia, the rodents were placed in a stereotactic head frame (Stoelting, IL, USA). Injections were performed with a 30-gauge needle and a 10 μL Hamilton syringe. Animals were injected with 3 μL of the desired vector combination. Vectors were diluted and normalized to each other. The rAAV2/7 vector encoding A53T α-SYN or eGFP was normalized to 9.0E11 GC/mL and rAAV2/8 vector encoding for either hMD3q-mCherry or mCherry was normalized to 3E10 GC/mL. When 2 viral vectors were injected together, they were injected simultaneously in a total volume of 3 μL containing the 2 vectors at the final concentrations cited above. Stereotactic coordinates used for the SN were anteroposterior, − 5.3; lateral, − 2.0; and dorsoventral, − 7.2 calculated from the dura using bregma as reference. The injection rate was 0.25 μL/min and the needle was left in place for an additional 5 min before being retracted. One week after stereotactic injection, we started the chronic stimulation by administering daily (5 days a week) Clozapine-N-Oxide (CNO) dihydrochloride (1 mg/kg diluted in saline, i.p., catalog #6329; Tocris Bioscience). Animals were sacrificed after behavioral analysis 4 or 12 weeks post-stereotactic injection.

### Rotarod test

The motor performance was assessed using mouse/rat Rotarod Treadmill (IITC Life Science, Woodland Hills, CA, USA). First, the animals were trained one week before the test was performed on 3 non-consecutive days. The training consisted of 3 trials of 4 min with at least 5 min of rest between the trials with an initial constant speed of 4 rpm; after 2 min, the rotational speed of the test was increased to 12 rpm. After this initial training, the rats were subjected to the test using an increasing speed from 4 to 40 rpm over a 100-s period, and the latency to fall was recorded.

### Cylinder test

The cylinder test was used to quantify forelimb use. Contacts made by each forepaw with the wall of 20-cm-wide clear glass cylinder were scored from the videotapes by an observer blinded to the animal’s identity. A total of 30 contacts (with fully extended digits executed with both forelimbs) were recorded for each animal. The number of impaired forelimb contacts was expressed as a percentage of total forelimb contacts. Non-lesioned control rats should score around 50% in this test.

### Electrophysiological recordings

Electrophysiological recordings were performed on acute brain slices from adult (10–12 weeks) female Wistar rats, 2 to 4 weeks after stereotactic vector injection (rAAV2/8 hM3Dq-mCherry vector or rAAV2/8 mCherry vector). Animals were anesthetized with isoflurane and decapitated using a guillotine. The brain was rapidly extracted and placed in ice-cold carboxygenated (95% O_2_ and 5% CO_2_) artificial cerebrospinal fluid (C-aCSF) cutting medium containing (in mM): 126 NaCl, 2.5 KCl, 1.2 NaH_2_PO_4_, 1.2 MgCl_2_, 2.4 CaCl_2_, 21.4 NaHCO_3_, 11.1 D-Glucose, and 1.25 kynurenic acid. The brains were mounted on a vibrating microtome (Leica VT1200S). Horizontal slices (150 μm) containing the substantia nigra pars compacta (SNpc) were collected and allowed to recover for at least 1 h at 35–36 °C in continuously carboxygenated C-aCSF. After recovery, slices were held in a recording chamber, maintained at 35–36 °C and perfused at a rate of 1.5–2 mL/min with carboxygenated aCSF containing (in mM): 126 NaCl, 2.5 KCl, 1.2 NaH_2_PO_4_, 1.2 MgCl_2_, 2.4 CaCl_2_, 21.4 NaHCO_3_, and 11.1 D-Glucose. Identification of putative DN in the SNpc was performed visually as large neurons near the medial terminal nucleus of the accessory optic tract. In addition, the following electrophysiological properties were checked to be qualified as DA neuron: spontaneous pacemaker firing between 1 and 5 Hz and wide extracellular spike waveforms. Spontaneous pacemaker firing was recorded as capacitive currents using the loose cell attached method described by Branch and Beckstead [51]. Recording borosilicate-glass pipettes (Hilgenberg), with a resistance of 3.5–5 MΩ, were made with a P-1000 puller (Sutter Instrument) and filled with a Na-HEPES-based solution (plus 20 mM NaCl, 290 mOsm, pH 7.40). Once the cell was identified, basal activity was recorded for 5 min. Thereafter effects of bath-applied CNO (40 μM) on DN pacemaking activity was determined for another 5 min. Analysis of frequency was calculated for a time frame of 2 min. In basal condition we took the last 2 min of recordings, while for the CNO condition we started 2 min after CNO administration. All recordings were acquired with Axopatch 200B amplifier (Clampex 10.0 software, Molecular Devices). Data was analyzed using pClamp software package (Axon Instruments), version 9.2.

### Immunohistochemical stainings

For histological analysis, we sacrificed the rats with sodium pentobarbital (200 mg/kg, i.p., Dolethal, Vetoquinol, Belgium). Intracardial perfusion with saline was followed by 4% paraformaldehyde (PFA) in phosphate buffered saline (PBS). After post-fixation overnight in 4% PFA, 50 μm-thick coronal brain sections were made with a vibrating microtome (HM 650 V, Microm, Germany). Prior immunostaining, antigen retrieval was performed using citrate buffer at 80 °C for 30 min followed by 20 min on ice. Immunohistochemistry (IHC) was performed on free-floating sections. Blocking was done in PBS-0.1% triton X-100, 10% goat serum for 30 min. Primary antibodies against tyrosine hydroxylase (TH, AB152, Millipore) and c-FOS (sc-52, Millipore) were used. As secondary antibody we used biotinylated anti-rabbit IgG (1:300 DakoCytomation), followed by the incubation with streptavidine-horseradish peroxidase complex (1:1000, DakoCytomation). c-FOS immunoreactivity was visualized using 3,3-diaminobenzidine (0.4 mg/mL, Sigma-Aldrich) and TH immunoreactivity was visualized using Vector SG (SK-4700, Vector Laboratories, CA) as a chromogen.

For fluorescent double or triple stainings, sections were rinsed 3 times in PBS, blocked in PBS-0.1% triton X-100 + 10% donkey serum for 30 min and then incubated overnight in PBS-0.1% triton X-100 + 10% donkey serum with the following antibodies: chicken anti-Red Fluorescent Protein (RFP, 1:1000, Rockland, Cat.N: 600–901-379S), rabbit anti-TH (1:5000, AB152, Millipore) and mouse anti P-S129 α-SYN (1:5000,11A5, Elan Pharmaceuticals). After one rinse and 4 washes of 5 min in PBS-0.1% triton X-100, the sections were incubated in the dark for 2 h in fluorochrome-conjugated secondary antibodies: donkey anti-mouse Alexa 488 (1:500, Molecular Probes, Invitrogen, Belgium), donkey anti-chicken Cy3 and goat anti-mouse Alexa 647 (1:500, Molecular Probes, Invitrogen). After one rinse and four washes of 5 min, the sections were rinsed in PBS and AD and mounted. The sections were coverslipped with mowiol containing DAPI (1:500). Staining was visualized using the Leica DM6 B automated upright microscope and images were taken using the Leica DFC7000 T camera. Fluorescent staining was visualized by confocal microscopy with a Leica Zeiss LSM 880 – Airyscan (Cell and Tissue Imaging Cluster (CIC), Supported by Hercules AKUL/15/37_GOH1816N and FWO G.0929.15 to Pieter Vanden Berghe, KU Leuven).

### Stereological quantification

The number of TH-positive cells in the SN was determined by stereological measurements using the Optical fractionator method in a computerized system as described before [52] (StereoInvestigator; MicroBrightField, Magdeburg, Germany). Every fifth section throughout the entire SN was analyzed, with a total of 7 sections for each animal. The coefficient of error calculated according to the procedure of Schmitz and Hof (Schmitz and Hof, 2005), varied between 0.05 and 0.10. For the fluorescent triple stereological quantifications, we performed similar stereological measurements, using the same parameters mentioned above but we made use of the software Stereologer®, SRC Biosciences (Stereology Resource Center, Inc.). We quantified both the injected and non-injected SN (internal control). An investigator blinded to the different groups performed all the analyses. For the analysis of TH loss in the STR we selected every 10th section throughout the entire STR with a total of 7 sections. We used FIJI software to set a threshold equal for all the sections. Threshold was set so that the cortex was zero. The dorsal STR was outlined and we quantified the percentage of positive area, which was set in reference to the non-injected side and represented as % striatal lesion.

### Sarkosyl extraction of the insoluble α-SYN fraction

Unfixed brains were dissected to isolate the SN and STR from both hemispheres, snap frozen in liquid nitrogen and kept at − 80 °C until extraction was performed. Brain samples were weighed and homogenized at 10% (w/v) in PBS buffer supplemented with phosphatase inhibitor cocktail (PhosSTOP™, Sigma-Aldrich) and protease inhibitors (Roche cOmplete EDTA free). Douncing and sonication (twice for 15 s each, using the following parameters: duty cycle: 30%, output: 2–3 using the BRANSON Sonifier 250) was used to disrupt and homogenize the tissue. Sarkosyl was added to 1%. 250 μl of sample was centrifuged at 6000 *g* for 10 min to remove cell debris. The supernatants (200 μl) were further centrifuged at 200,000 *g* for 60 min at 20 °C in an Optima TLA (120.2) Ultracentrifuge (Beckman). The supernatant was considered the soluble fraction. The pellets were washed with PBS-1% Sarkosyl and resuspended in 200 μl of PBS buffer, considering the insoluble fraction.

### Western blot analysis

For western blot analysis, 20 μg of protein for the total and soluble fractions was mixed with a 6x denaturating buffer (50 mM Tris-HCl, pH 6.8, 4% SDS, 2% β-mercaptoethanol, 12% glycerol and 0.01% bromophenol blue). The insoluble fraction was mixed with the denaturating buffer using the same volume for all conditions. Samples were heated to 95 °C for 10 min. Samples were separated using 4–20% Tris-Glycine gradient gels (Bio-Rad). Separated proteins were transferred to a polyvinylidene fluoride (PVDF) membrane (Bio-Rad). Proteins were fixed to the PVDF membrane using 0.4% PFA for 20 min, thereafter non-specific binding sites were blocked for 15 min in PBS with 0.1% Triton X-100 (PBS-T) and 5% non-fat milk. After overnight incubation at 4 °C with primary antibodies: rabbit anti-TH (MAB318, Chemicon), mouse anti-vinculin (V9131, sigma), mouse anti-P-S129 α-SYN (11A5, Elan Pharmaceuticals), mouse anti-α-SYN (4B12, Thermo scientific), rabbit anti-synaptophysin (YE269, abcam), rabbit anti-PSD95 (ab18258, abcam) and mouse anti-synaptobrevin (104,211, synaptic systems), membranes were washed 3 times with PBS-T and incubated with horseradish peroxidase-conjugated secondary antibody (either goat anti-mouse or anti-rabbit depending of the primary antibody used (Dako, Glostrup)) for 1 h. After 3 washing steps the proteins were visualized using enhanced chemiluminescence (ECL prime Amersham GE healthcare) and quantified using the software ImageQuant™ TL software (GE Heathcare).

### Statistics

Graph creation and statistical analysis was performed using Graphpad Prism for Windows (GraphPad software Inc.) version 8.0.0. Results are presented as means ± standard deviation. Normality of data was tested using the Shapiro-Wilk test. Statistical significance was assessed using either a T-Test or, when multiple groups were analyzed simultaneously, one-way or two-way ANOVA followed by post-hoc Bonferroni multiple comparison test was used. Significance was represented as follows: *p < 0.05, **p < 0.01, *** *p* < 0.001 and ****p < 0.0001.

## Results

### Validation of DREADDs expression and neuronal activity modulation in vivo

**Fig. 1:**
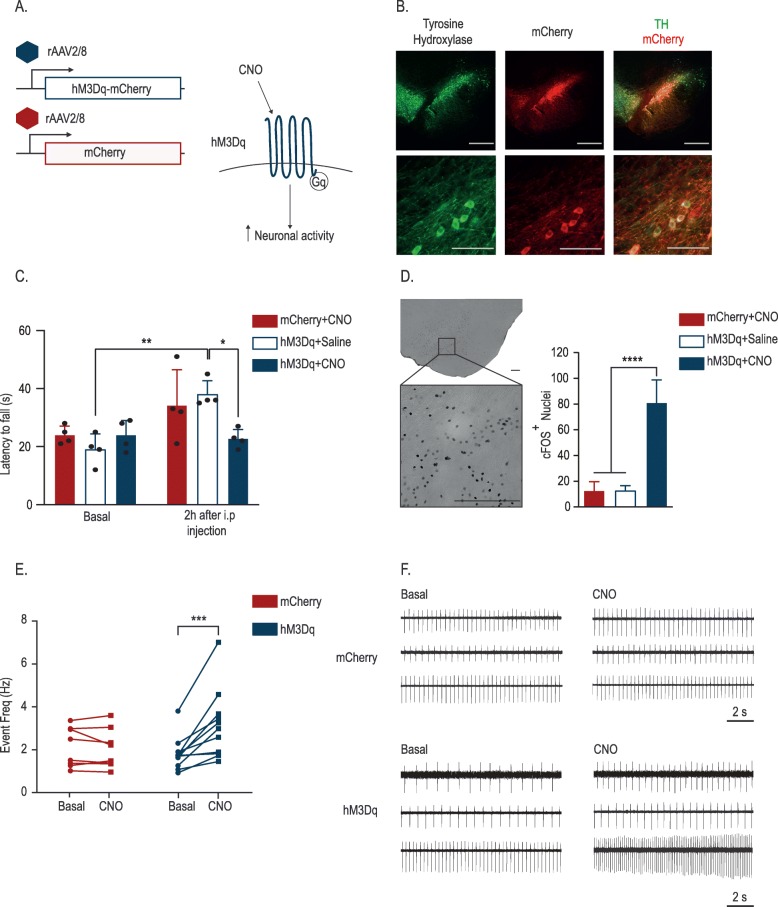
Validation of neuronal activity modulation using DREADDs. **a** Schematic view of an activating DREADD (hM3Dq) receptor. **b** rAAV-mediated overexpression of the hM3Dq receptor in dopaminergic nigral neurons assessed by IHC staining. Tyrosine Hydroxylase (TH), and mCherry were used to localize the expression of the transgene in the DN (scale bars = 500 μm (top panel) and 100 μm (bottom panel). **c** Latency to fall from the accelerating rotarod in basal condition and after administration of CNO (1 mg/kg) or saline in rAAV2/8 hM3Dq or rAAV2/8 mCherry vector-injected animals (Mean ± SD, *p < 0.05, **p < 0.01 by two-way ANOVA and Bonferroni posthoc test, *n* = 4). **d** Representative image of hM3Dq injected animal treated with CNO and quantification of the cFOS+ nuclei in the different experimental groups (scale bar = 250 μm) (Mean ± SD, ****p < 0.0001 by one-way ANOVA and Bonferroni posthoc test, n = 4). **e** Firing frequency of putative DN from animals overexpressing the hM3Dq receptor or mCherry in basal condition and after CNO administration. (*n* = 8 to 11 DNs from at least 5 different animals, two-way ANOVA Bonferroni posthoc test ***p < 0.0005). **f** Representative traces from 6 putative DN (overexpressing mCherry or the hM3Dq-mCherry receptor, in basal condition and post-CNO administration)

In order to modulate neuronal activity in rat nigral DN, we used DREADDs technology [47]. These modified GPCR receptors are inactive under basal conditions and only activated when the ligand CNO is administered. To express these receptors in vivo we stereotactically injected a recombinant adeno-associated viral vector serotype 2/8 (rAAV2/8) encoding the activating DREADDs (hM3Dq) fused to mCherry under the control of the CamKII promotor in the rat SN. As control, we used an equal dose of a rAAV2/8 vector encoding mCherry (Fig. 1A). First, we assessed the expression of the hM3Dq receptor throughout the SNpc by colocalization of mCherry and tyrosine hydroxylase (TH), a marker for DN. Fifteen days after injection, the majority (> 85%) of the nigral DN was efficiently transduced (Additional file 1: Figure S1). Prominent mCherry expression was detected in the cell bodies and axons of the DN in the SN (Fig. 1B**)**. Next, to validate successful modulation of neuronal activity of the DN in the rat SNpc, we first assessed motor performance. We performed the rotarod test on animals injected with either the rAAV2/8 hM3Dq or the mCherry control vector in basal conditions and after saline or CNO treatment (*n* = 4/group). In basal conditions, we did not observe any differences in the latency to fall between the different groups. Two hours after treatment with CNO or saline, both control groups, mCherry + CNO and hM3Dq + saline improved their performance compared to the first test, probably due to a learning effect. In contrast, the experimental hM3Dq + CNO group did not improve and presented a significantly lower latency to fall compared to the control groups (Fig. 1C), indicating that neuronal modulation impaired the motor performance. Motor changes after neuromodulation in the dopaminergic system have already been described. Bilateral expression of hM3Dq in the SN DN resulted in hyperactivity [53]. In our case, we hypothesize that acute asymmetrical dopamine release in the STR provoked the motor deficits. Second, we performed IHC staining for the immediate early gene product cFOS, which is considered a marker for cellular activation [54]. In the experimental hM3Dq group treated with CNO, we observed a significant increase in the number of cFos-positive nuclei in the SN compared to the saline-treated animals or the mCherry control group (Fig. 1D). Third, we performed electrophysiological recordings on putative nigral DN of ex vivo brain slices. In basal conditions, the recorded nigral neurons presented a slow (1–5 Hz) regular pacemaking activity typical for DN (Fig. 1F) [55]. Administration of CNO to the brain slices resulted in a significant increase (average fold increase from basal condition was 1,73) in firing rate in the hM3Dq group indicating increased neuronal activity. In mCherry control animals, the firing rate remained unaffected (Fig. 1E,F).

### Chronic neuronal activity modulation aggravates α-SYN induced behavioral deficits without modifying dopaminergic neuronal loss

**Fig. 2:**
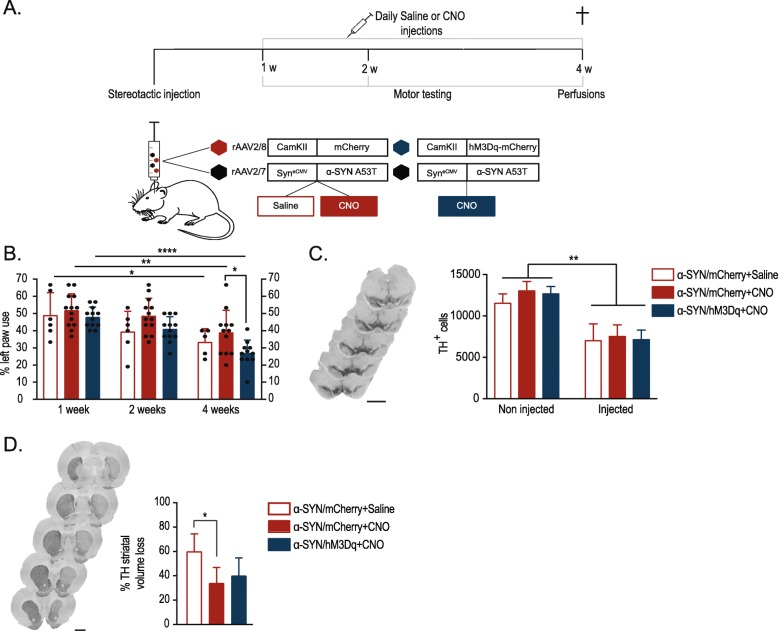
Chronic neuronal activity modulation in the SN aggravates α-SYN induced behavioral deficits without affecting dopaminergic neuronal loss. **a** Schematic representation of experimental set-up. **b** Detection of unilateral motor deficits using the cylinder test at different time points p.i (*n* = 5 to 13, Mean ± SD, two-way ANOVA Bonferroni Multiple Comparison test, *p < 0.05, **p < 0.01, ***p < 0.0001). **c** Representative images of TH staining in the midbrain (scale bar 1 mm) and stereological quantifications of DN in the injected and non-injected SNpc (n = 5 to 6, Mean ± SD, one-way ANOVA Bonferroni Multiple Comparison test, **p < 0.01). **d** Representative images of TH staining in the striatum (scale bar 2 mm). Striatal volume loss was calculated using FIJI software, in relation to the non-injected side (n = 5 to 6, Mean ± SD, one-way ANOVA Bonferroni Multiple Comparison test, *p < 0.05)

Next, we studied the effect of chronic synaptic modulation on α-SYN-induced behavioral deficits, neurotoxicity, α-SYN spreading and pathology in a rat model based on rAAV2/7 vector-mediated A53T α-SYN overexpression [46]. To do this, a group of animals was injected with the rAAV2/7 α-SYN vector combined with the rAAV2/8 hM3Dq vector and treated daily for three weeks with CNO (Fig. 2A). Both vectors efficiently transduced SN DN (Additional file 1: Figure S1). As control, we injected two groups of animals unilaterally with the rAAV2/7 α-SYN vector combined with the rAAV2/8 mCherry vector and treated one group with CNO and the other one with saline. In order to follow up motor deficits we subjected the animals to the cylinder test at 1, 2 or 4 weeks post injection (p.i). One should take into account that here the cylinder test was performed 24 h after CNO injection, when CNO is not expected to be active anymore. In all groups, we found a significant progressive decrease of the left paw use, induced by the unilateral overexpression of α-SYN. Four weeks p.i, the motor deficits were more pronounced in the CNO-treated rAAV α-SYN/hM3Dq vector group compared to the CNO-treated rAAV α-SYN/mCherry vector control animals (Fig. 2B). In order to rule out any non-specific effect of protein overexpression or CNO treatment we also combined rAAV2/7 GFP vector overexpression with the rAAV2/8 hM3Dq vector. No significant changes in behavior nor in weight were observed due to the CNO treatment (Additional file 1: Figure S2), clearly indicating that the enhanced behavioral deficits were caused by chronic modulation of neuronal activity in the α-SYN-overexpressing neurons.

Subsequently, we assessed whether chronic neuromodulation also affected neuronal cell loss. IHC analysis revealed a 50% loss of TH+ nigral neurons upon overexpression of α-SYN in all groups, without significant differences after chronic modulation (Fig. 2C). Further, we determined the loss of TH+ terminals in the striatum. Again, we found no difference in striatal lesion between the rAAV α-SYN/hM3Dq and the rAAV α-SYN/mCherry CNO vector-treated animals (Fig. 2D). Somewhat unexpectedly, we observed a smaller striatal lesion in both CNO-treated groups, compared to the α-SYN/mCherry saline-treated animals.

### Chronic neuronal activity modulation induces changes in α-synucleinopathy in vivo

**Fig. 3:**
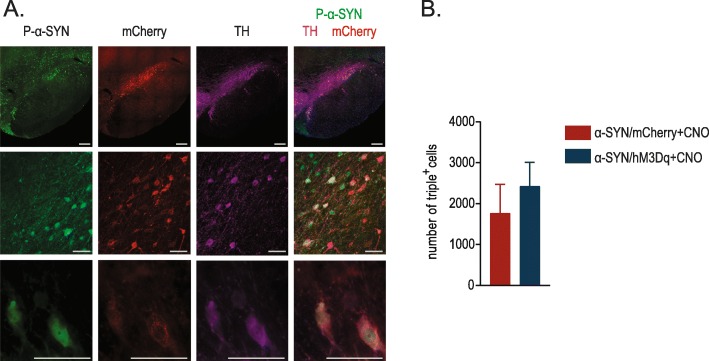
Stereological quantifications of the number of P-S129 α-SYN, mCherry and TH+ cells. **a** Confocal images of triple immunofluorescent staining for P-S129 α-SYN (green), mCherry (red) and TH (purple) in the injected SN at different magnifications (Scale bars = 250 μm upper panel and 50 μm lower and middle panel). **b** Quantifications of P-S129 α-SYN/TH/mCherry triple positive cells. (n = 5–6, Mean ± SD, Unpaired T-test)

Alpha-SYN aggregation is considered an important hallmark of PD pathology. To assess whether chronic neuronal modulation affects α-SYN pathology we stereologically quantified the number of P-S129 α-SYN-positive DN (P-S129 α-SYN) in the SN (Fig. 3A). We observed no significant difference in the total number of P-S129 α-SYN/TH/mCherry triple positive cells between the activated and non-activated α-SYN overexpressing animals (Fig. 3B). In addition, we performed P-S129 α-SYN staining in different brain regions to study potential spreading due to the increase in neuronal activity. However, we did not find P-S129 α-SYN deposits in regions outside of the SN and STR.

**Fig. 4:**
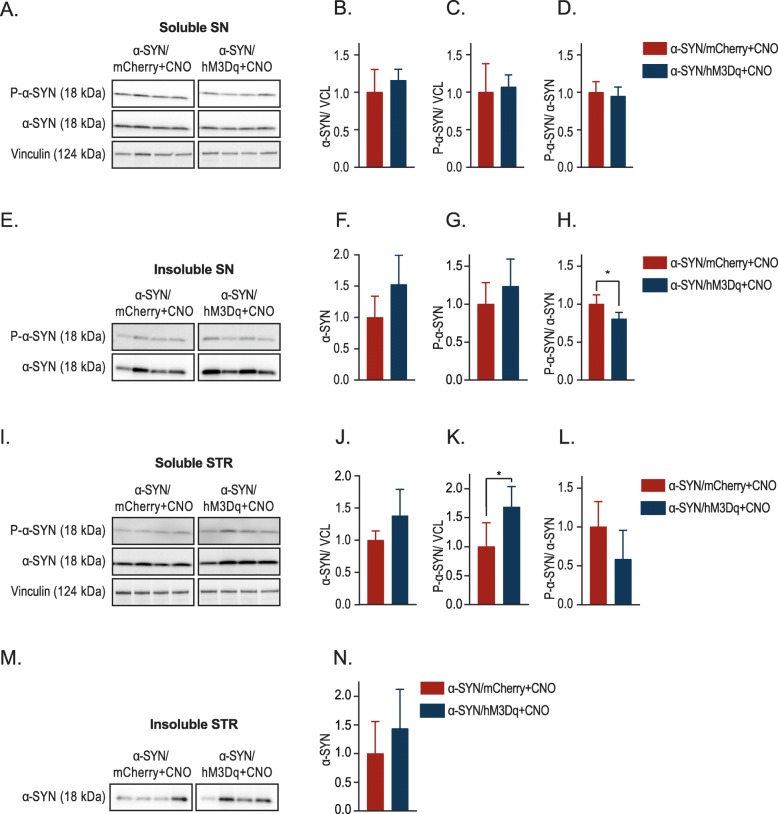
Effect of chronic neuronal activation on α-SYN localisation and phosphorylation levels. **a** Representative western blot of the nigral sarkosyl soluble fraction. **b** - **d** Quantifications of the nigral soluble fraction either for α-SYN relative to the loading control vinculin (VCL) (**b**), P-S129 α-SYN relative to vinculin (VCL)(**c**) or the P-S129 α-SYN/total-α-SYN ratio (**d**) (*n* = 6, Mean ± SD, Unpaired T-Test). **e** Representative western blot of the nigral sarkosyl insoluble fraction. **f**-**h** Quantifications from the nigral insoluble fractions either for total α-SYN (**f**), P-S129 α-SYN (**g**) or the P-S129 α-SYN/total α-SYN ratio (**h**) (n = 4, Mean ± SD, Unpaired T-Test *p < 0.05). **i** Representative western blot of the striatal soluble fraction. **j - l**. Quantifications of the striatal soluble fractions either for α-SYN relative vinculin (VCL)(**j**), P-S129 α-SYN relative to vinculin (VCL)(**k**) or the P-S129 α-SYN/total α-SYN ratio (**l**) (n = 6, Mean ± SD, Unpaired T-Test *p < 0.05). **m** Representative western blot of the striatal sarkosyl insoluble fraction. **n** Quantification from the striatal insoluble fraction for total α-SYN. In all graphs the results have been normalized to mCherry/α-SYN+CNO control, which has been artificially set to 1 (n = 4, Mean ± SD, Unpaired T-Test)

To characterize more in detail the effect of neuronal modulation on α-SYN pathology, we performed biochemical analysis of the injected SN as well as the connecting STR. We performed a sarkosyl extraction on the SN and separated the sarkosyl soluble and insoluble fraction. The sarkosyl insoluble fraction consists of aggregated or fibrillar α-SYN, while the soluble fraction contains mostly monomeric or small oligomeric α-SYN species. When analyzing the nigral sarkosyl soluble fractions we observed that chronic neuronal activation (α-SYN /hM3Dq + CNO) did not alter total α-SYN levels or phosphorylated α-SYN levels, resulting in an unaltered P-S129 α-SYN/α-SYN ratio compared to the α-SYN/mCherry controls (Fig. 4 A, B, C and D). When analyzing the sarkosyl insoluble fractions, we observed a trend towards higher levels of total α-SYN and P-S129 α-SYN levels upon chronic stimulation. The P-S129 α-SYN/α-SYN ratio though decreases with stimulation (Fig. 4 E, F, G and H).

Next, we analyzed the connecting STR. Chronic neuronal activation induced a trend towards higher levels of total α-SYN and significantly higher levels of P-S129 α-SYN in the soluble fractions (Fig. 4 I, J and K). Further, we detected a trend toward a higher P-S129 α-SYN/α-SYN ratio (Fig. 4 I, L). Analogously, in the insoluble striatal fractions we observed a trend towards higher levels of total α-SYN in the STR (Fig. 4 M, N). The levels of P-S129 α-SYN were under the detection limit. Overall, these results indicate that neuronal activation can modify α-SYN localization as well as α-SYN phosphorylation. These changes could alter the toxic properties of α-SYN in the cell, or more importantly at the level of the synapse.

### Chronic neuronal activation modulates synaptic proteins in the STR

**Fig. 5:**
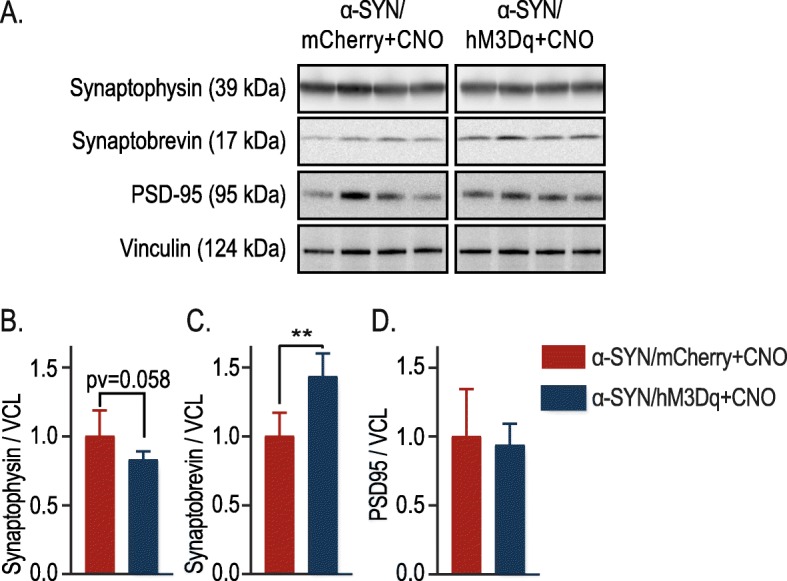
Chronic neuronal activation induces changes in synaptic proteins in the striatum. **a** Representative western blots of the total striatal fraction for the presynaptic proteins (synaptophysin and synaptobrevin), the postsynaptic protein (PSD-95) and loading control vinculin (VCL). **b - d** Quantifications of the western blot: Synaptophysin relative to loading control vinculin (VCL) (**b**), Synaptobrevin relative to loading control vinculin (VCL) (**c**) or PSD-95 relative to loading control vinculin (VCL) (**d**). In all graphs the results have been normalized to mCherry/α-SYN + CNO control, which has been artificially set to 1 (n = 6, Mean ± SD, Unpaired T-Test, **p < 0.01)

Based on our biochemistry data, we hypothesized that this increase in total soluble and insoluble α-SYN in the STR might induce synaptic changes accountable for the enhanced motor deficits we observed upon chronic modulation. To test this hypothesis, we analyzed different synaptic proteins (Fig. 5). We found slightly decreased levels of synaptophysin and significantly increased levels of synaptobrevin (both markers for presynaptic terminals) in the STR upon chronic modulation (Fig. 5 A, B and C). On the contrary, we found no changes in the postsynaptic protein PSD-95 (Fig. 5 A, D), which is present in excitatory synapses co-localizing with DA receptors in tripartite synapses [56, 57].

## Discussion

In this study, we used DREADDs technology to modulate neuronal activity and assessed the effect in an α-SYN-based model for PD. First, we demonstrated that we could successfully increase neuronal activity in nigral DN via viral vector-mediated overexpression of hM3Dq and CNO administration. Using motor testing, IHC as well as ex vivo electrophysiology we have extensively validated the neuronal activation in DN in vivo.

Next, we applied this technology in our viral vector-based rat model to study the effect on α-SYN pathology and toxicity. Chronic stimulation via optogenetic or chemogenetic techniques has never been used to study α-SYN pathology in the context of PD. We found that chronic neuronal activation aggravated α-SYN-induced motor deficits in vivo. Interestingly, these behavioral changes were not accompanied by increased nigral dopaminergic cell loss nor by changes in the number of phosphorylated nigral neurons (Fig. 3). Using biochemical techniques, we observed that chronic neuronal activation leads to increased levels of insoluble α-SYN and P-S129 α-SYN in the STR and a reduced ratio of P-S129 α-SYN/total α-SYN in the SN. We therefore conclude that increasing neuronal activity in DN promotes striatal enrichment of the phosphorylated form of α-SYN. Further, we observed changes in synaptophysin and synaptobrevin, two presynaptic markers, but not in postsynaptic protein PSD-95. At this moment we cannot explain why we observe differential effects on these proteins. They both localize in the synaptic terminal and interact with each other in the synaptic vesicles (SV) as well as in the plasma membrane and dissociate during stimulation [58]. It is uncertain how this interaction works, Gordon et al. propose a role for synaptophysin during SV recycling, retrieving synaptobrevin during SV endocytosis [59]. The decrease in synaptophysin points to a decrease in synaptic density, its expression has been linked both to DA release as well as to motor performance in a mouse model for PD [60, 61]. We postulate that a higher α-SYN load at the level of the synapse induces presynaptic alterations, resulting in aggravated motor dysfunction.

Synaptic activity modulation has been applied before in other neurodegenerative diseases. In Alzheimer’s disease (AD) several studies have used optogenetic and chemogenetic tools to study the effect on disease pathology and progression. Using diverse approaches they reveal that synaptic modulation can regulate the secretion, the conformation and the spreading of Aβ-plaques and Tau [62–68]. More recently Schultz and colleagues observed extensive Tau spreading toward different brain regions when modulating synaptic activity via DREADDs technology [68]. In our study, we could observe enrichment of P-S129 α-SYN at terminals in the STR, but no trans-synaptic spreading toward different brain regions in this time frame [46]. In contrast to α-SYN, rAAV-mediated Tau overexpression results in Tau spreading towards synaptically connected regions. Despite the fact that these proteins are often pooled together and share some traits, it is important to acknowledge the differences in intrinsic properties, which might be responsible for the differences we observe. One approach to enhance spreading in our model is to combine the viral vector approach with recombinant α-SYN fibrils as we pioneered recently [3].

Chemogenetic tools have been used in other PD models [69–73]. Acute stimulation of cholinergic pedunculopontine neurons has been shown to improve motor deficits [74]. More recently, Stanojlovic et al. showed cognitive improvement after acute modulation of the orexin neurons in the medial pre-frontal cortex [75]. DREADDs expression was shown effective in modulating the DA secretion levels in transplanted induced DN [70, 73]. Whilst others have shown improvement of motor deficits after acute stimulation in transplanted DN, we show that chronic stimulation of nigral DN results in a decline of motor function. Although the experimental set-up is different, our data might be relevant when considering neuronal stimulation as a therapeutic approach. Acute treatment might result in temporary beneficial effects but chronic treatment could induce antagonistic effects. Deep brain stimulation (DBS) is another way of modulating synaptic activity. DBS in the subthalamic nucleus or globus pallidus is performed routinely with remarkable beneficial symptomatic effects for a large number of PD patients. Based on our results, it would be interesting to study the effect of DBS on α-SYN pathology as this might promote α-SYN relocalization.

Of note, during this project we encountered an unexpected effect of the DREADDs technology. Although CNO was considered to be an inert ligand [47], CNO-treated control animals presented a smaller striatal lesion after α-SYN overexpression (Fig. 2D). This DREADD-independent CNO effect has also been noticed by Schultz et al. where they suggest a CNO-based neuroprotection in their viral vector-based Tau model [68]. Remarkably, in another report unrelated to DREADDs technology, Jiang and colleagues evaluate different metabolites of clozapine and highlight the neuroprotective role of CNO through the inhibition of a microglial pathway [76]. An alternative explanation could be the conversion of CNO to its precursor clozapine, which recently has been shown to be an even stronger activator of the DREADDs receptors [77–79]. Alternatively, clozapine could be used instead of CNO as a DREADDs activator, when applied in subtherapeutic doses. Our findings highlight the importance of including the proper controls when using DREADDs technology.

## Conclusion

In conclusion, our study demonstrates successful chronic stimulation of nigral rat DN using DREADDs technology. We are the first to use this technology to study the effect of neuronal modulation on α-SYN pathology in vivo. We found that chronic neuronal modulation aggravates α-SYN induced motor deficits. In addition, it induces an enrichment of phosphorylated and insoluble α-SYN in dopaminergic striatal terminals, along with changes in synaptic markers. Overall, our data link synaptic activity and α-SYN-induced motor deficits and pathogenesis. Future studies may investigate whether chronic decrease of neuronal activity could inhibit α-SYN spreading in the rodent brain.

## Supplementary information

**Additional file 1: Figure S1.** Transduction efficiency of rAAV2/7 α-SYN and rAAV2/8 hM3Dq vector (tagged with mCherry tag) in DN. **Figure S2.** Chronic neuronal activity modulation in rAAV2/7 GFP vector injected animals does not affect motor behavior or TH+ cell numbers. **Figure S3.** Complete and uncropped western blots from Fig. 4. **Figure S4.** Complete and uncropped western blots from Fig. 5.

## Data Availability

The datasets used and/or analysed during the current study are in the KU LEUVEN network drive and available from the corresponding author on reasonable request.

## Abbreviations

aCSF: Artificial cerebrospinal fluid
AD: Alzheimer’s disease
CNO: Clozapine-N-oxide
DBS: Deep brain stimulation
DLB: Dementia with Lewy bodies
DREADDs: Designer Receptors which are Exclusively Activated by Designer Drugs
eGFP: Enhanced green fluorescent protein
GC: Genomic copies
GCI: Glial cytoplasmic inclusions
GPCRs: G-protein coupled receptors
i.p: Intraperitoneal
LB: Lewy bodies
MSA: Multiple system atrophy
MSN: Medium spiny neurons
p.i: Post injection
PBS: Phosphate buffered saline
PD: Parkinson’s disease
PFA: Paraformaldehyde
p-S129 α-SYN: Phosphorylation of alpha-synuclein at serine 129
rAAV: Recombinant adeno-associated virus
SN: Substantia nigra
SNpc: Substantia nigra pars compacta
STR: Striatum
SV: Synaptic vesicles
TH: Tyrosine hydroxylase
VCL: Vinculin
α-SYN: Alpha-synuclein

## References

[1] Spillantini MG, Schmidt ML, Lee VM, Trojanowski JQ, Jakes R, Goedert M (1997). Alpha-synuclein in Lewy bodies. Nature.

[2] Goedert Michel, Spillantini Maria Grazia, Del Tredici Kelly, Braak Heiko (2012). 100 years of Lewy pathology. Nature Reviews Neurology.

[3] Peelaerts W, Bousset L, Van der Perren A, Moskalyuk A, Pulizzi R, Giugliano M (2015). α-Synuclein strains cause distinct synucleinopathies after local and systemic administration. Nature.

[4] McCann H, Stevens CH, Cartwright H, Halliday GM (2014). α-Synucleinopathy phenotypes. Park Relat Disord. Elsevier Ltd.

[5] Maroteaux LJTC, Synuclein SRH (1988). A Neuron-Specific Protein Localized to the Nucleus and Presynaptic Nerve Terminal. J Neurosci.

[6] Uéda K, Fukushima H, Masliah E, Xia Y, Iwai A, Yoshimoto M (1993). Molecular cloning of cDNA encoding an unrecognized component of amyloid in Alzheimer disease. Proc Natl Acad Sci U S A. National Academy of Sciences.

[7] Burré J, Sharma M, Tsetsenis T, Buchman V, Etherton MR ST. Alpha-Synuclein Promotes SNARE-Complex Assembly in vivo and in vitro. Science (80- ). 2010;24:14 10.1126/science.1195227 PMC3235365 20798282

[8] Diao J, Burré J, Vivona S, Cipriano DJ, Sharma M, Kyoung M (2013). Native α-synuclein induces clustering of synaptic-vesicle mimics via binding to phospholipids and synaptobrevin-2/VAMP2. Elife.

[9] Burré J (2015). The synaptic function of α-Synuclein. J Parkinsons Dis IOS Press.

[10] Chandra Sreeganga, Gallardo Gilbert, Fernández-Chacón Rafael, Schlüter Oliver M., Südhof Thomas C. (2005). α-Synuclein Cooperates with CSPα in Preventing Neurodegeneration. Cell.

[11] Cabin DE, Shimazu K, Murphy D, Cole NB, Gottschalk W, McIlwain KL (2002). Synaptic vesicle depletion correlates with attenuated synaptic responses to prolonged repetitive stimulation in mice lacking alpha-synuclein. J Neurosci Society for Neuroscience.

[12] Nemani VM, Lu W, Berge V, Nakamura K, Onoa B, Lee MK (2010). Increased expression of α-Synuclein reduces neurotransmitter release by inhibiting synaptic vesicle Reclustering after endocytosis. Neuron Elsevier Ltd.

[13] Gaugler MN, Genc O, Bobela W, Mohanna S, Ardah MT, El-Agnaf OM (2012). Nigrostriatal overabundance of α-synuclein leads to decreased vesicle density and deficits in dopamine release that correlate with reduced motor activity. Acta Neuropathol Springer-Verlag.

[14] Uversky VN (2003). A protein-chameleon: conformational plasticity of alpha-synuclein, a disordered protein involved in neurodegenerative disorders. J Biomol Struct Dyn.

[15] Bartels T, Choi JG, Selkoe DJ (2011). α-Synuclein occurs physiologically as a helically folded tetramer that resists aggregation. Nature. Nat Publ Group.

[16] Fauvet B, Mbefo MK, Fares MB, Desobry C, Michael S, Ardah MT (2012). α-Synuclein in central nervous system and from erythrocytes, mammalian cells, and Escherichia coli exists predominantly as disordered monomer. J Biol Chem.

[17] Sultana Z, Paleologou KE, Al-Mansoori KM, Ardah MT, Singh N, Usmani S et al (2011) Dynamic modeling of α-synuclein aggregation in dopaminergic neuronal system indicates points of neuroprotective intervention: experimental validation with implications for Parkinson’s therapy. Neuroscience 10.1016/j.neuroscience.2011.10.018 22056602

[18] Bridi JC, Hirth F (2018). Mechanisms of α-Synuclein induced Synaptopathy in Parkinson’s disease. Front Neurosci.

[19] Conway KA, Lee SJ, Rochet JC, Ding TT, Williamson RE, Lansbury PT (2000). Acceleration of oligomerization, not fibrillization, is a shared property of both alpha-synuclein mutations linked to early-onset Parkinson’s disease: implications for pathogenesis and therapy. Proc Natl Acad Sci U S A.

[20] Winner B, Jappelli R (2011). In vivo demonstration that α-synuclein oligomers are toxic. Proc Natl Acad Sci.

[21] Bousset L, Pieri L, Ruiz-Arlandis G, Gath J, Jensen PH, Habenstein B (2013). Structural and functional characterization of two alpha-synuclein strains. Nat Commun.

[22] Candelise N, Schmitz M, Llorens F, Villar-Piqué A, Cramm M, Thom T (2019). Seeding variability of different alpha synuclein strains in synucleinopathies. Ann Neurol. John Wiley & Sons, Ltd.

[23] Freundt EC, Maynard N, Clancy EK, Roy S, Bousset L, Sourigues Y, Covert M, Ronald Melki K, Brahic KM (2012). Neuron-to-neuron transmission of α-synuclein fibrils through.pdf. Ann Neurol. Ann Neurol.

[24] Paleologou KE, Schmid AW, Rospigliosi CC, Kim H-Y, Lamberto GR, Fredenburg RA (2008). Phosphorylation at Ser-129 but not the phosphomimics S129E/D inhibits the fibrillation of alpha-synuclein. J Biol Chem American Society for Biochemistry and Molecular Biology.

[25] Wu B, Liu Q, Duan C, Li Y, Yu S, Chan P (2010). Phosphorylation of a-synuclein upregulates tyrosine hydroxylase activity in MN9D cells. Acta Histochem.

[26] Chen L, Feany MB (2005). A-Synuclein phosphorylation controls neurotoxicity and inclusion formation in a Drosophila model of Parkinson disease. Nat Neurosci. Nat Publ Group.

[27] Li JY, Englund E, Holton JL, Soulet D, Hagell P, Lees AJ (2008). Lewy bodies in grafted neurons in subjects with Parkinson’s disease suggest host-to-graft disease propagation. Nat Med.

[28] Kordower JH, Chu Y, Hauser RA, Freeman TB, Olanow CW (2008). Lewy body-like pathology in long-term embryonic nigral transplants in Parkinson’s disease. Nat Med.

[29] Braak Heiko, Ghebremedhin Estifanos, Rüb Udo, Bratzke Hansjürgen, Del Tredici Kelly (2004). Stages in the development of Parkinson’s disease-related pathology. Cell and Tissue Research.

[30] Braak H, Del Tredici K, Rüb U, de Vos RA, Jansen Steur EN (2003). BE. Staging of brain pathology related to sporadic Parkinson’s disease. Neurobiol Aging.

[31] Visanji NP, Brooks PL, Hazrati L-N, Lang AE (2013). The prion hypothesis in Parkinson’s disease: Braak to the future. Acta Neuropathol Commun.

[32] Desplats P, Lee H-J, Bae E-J, Patrick C, Rockenstein E, Crews L (2009). Inclusion formation and neuronal cell death through neuron-to-neuron transmission of alpha-synuclein. Proc Natl Acad Sci U S A.

[33] Lee SJ, Desplats P, Lee HJ, Spencer B, Masliah E (2012). Cell-to-cell transmission of α-synuclein aggregates. Methods Mol Biol.

[34] Burre J, Sharma M, Sudhof TC. Alpha-Synuclein assembles into higher-order multimers upon membrane binding to promote SNARE complex formation. Proc Natl Acad Sci U S A. 2014/09/24. 2014;111:E4274–E4283 10.1073/pnas.1416598111 PMC4210039 25246573

[35] Choi B-K, Choi M-G, Kim J-Y, Yang Y, Lai Y, Kweon D-H (2013). Large α-synuclein oligomers inhibit neuronal SNARE-mediated vesicle docking. Proc Natl Acad Sci U S A.

[36] Halbgebauer S, Öckl P, Wirth K, Steinacker P, Otto M (2016) Protein biomarkers in Parkinson’s disease: Focus on cerebrospinal fluid markers and synaptic proteins. Mov. Disord:848–860 10.1002/mds.26635 27134134

[37] Calabresi P, Standaert DG, Chiasserini D, Parnetti L (2016). Biomarkers in Parkinson’s disease: from pathophysiology to early diagnosis. Mov Disord. John Wiley & Sons, Ltd.

[38] Jin J, Hulette C, Wang Y, Zhang T, Pan C, Wadhwa R (2006). Proteomic identification of a stress protein, Mortalin/mthsp70/GRP75. Mol Cell Proteomics.

[39] Lundblad M, Decressac M, Mattsson B, Bjorklund A (2012). Impaired neurotransmission caused by overexpression of -synuclein in nigral dopamine neurons. Proc Natl Acad Sci.

[40] Janezic S, Threlfell S, Dodson PD, Dowie MJ, Taylor TN, Potgieter D (2013). Deficits in dopaminergic transmission precede neuron loss and dysfunction in a new Parkinson model. Proc Natl Acad Sci U S A.

[41] Subramaniam M, Althof D, Gispert S, Schwenk J, Auburger G, Kulik A (2014). Mutant α-Synuclein enhances firing frequencies in dopamine Substantia Nigra neurons by oxidative impairment of A-type potassium channels. J Neurosci.

[42] Micheva KD, Busse B, Weiler NC, O’Rourke N, Smith SJ (2010). Single-synapse analysis of a diverse synapse population: proteomic imaging methods and markers. Neuron Elsevier.

[43] Gardoni F, Bellone C (2015). Modulation of the glutamatergic transmission by dopamine: a focus on Parkinson, Huntington and addiction diseases. Front cell Neurosci. Frontiers.

[44] Picconi B, Piccoli G, Calabresi P. Synaptic dysfunction in Parkinson’s disease. Synaptic Plast. Springer, Vienna; 2012. p. 553–72 10.1007/978-3-7091-0932-8_24 22351072

[45] Villalba RM, Smith Y. Neuroglial plasticity at striatal glutamatergic synapses in Parkinson’s disease. Front Syst Neurosci. 2011;5:1–9. 10.3389/fnsys.2011.00068 PMC3159891 21897810

[46] Van der Perren A, Toelen J, Casteels C, Macchi F, Van Rompuy A-S, Sarre S (2014). Longitudinal follow-up and characterization of a robust rat model for Parkinson’s disease based on overexpression of alpha-synuclein with adeno-associated viral vectors. Neurobiol Aging Elsevier Inc.

[47] Armbruster BN, Li X, Pausch MH, Herlitze S, Roth BL (2007). Evolving the lock to fit the key to create a family of G protein-coupled receptors potently activated by an inert ligand. Proc Natl Acad Sci U S A.

[48] Urban DJ, Roth BL (2014). DREADDs (designer receptors exclusively activated by designer drugs): Chemogenetic tools with therapeutic utility. Annu Rev Pharmacol Toxicol.

[49] Roth Bryan L. (2016). DREADDs for Neuroscientists. Neuron.

[50] Van der Perren A, Toelen J, Carlon M, Van den Haute C, Coun F, Heeman B (2011). Efficient and stable transduction of dopaminergic neurons in rat substantia nigra by rAAV 2/1, 2/2, 2/5, 2/6.2, 2/7, 2/8 and 2/9. Gene Ther. Nat Publ Group.

[51] Branch SY, Beckstead MJ (2012). Methamphetamine produces bidirectional, concentration-dependent effects on dopamine neuron excitability and dopamine-mediated synaptic currents. J Neurophysiol American Physiological Society.

[52] Baekelandt V, Claeys A, Eggermont K, Lauwers E, De Strooper B, Nuttin B (2002). Characterization of Lentiviral vector-mediated gene transfer in adult mouse brain. Hum Gene Ther.

[53] Wang S, Tan Y, Zhang JE, Luo M (2013). Pharmacogenetic activation of midbrain dopaminergic neurons induces hyperactivity. Neurosci Bull.

[54] Dragunow M, Bag P (1989). The use of c-fos as a metabolic marker in neuronal pathway tracing. J Neurosci.

[55] Grace AA, Onn SP (1989). Morphology and electrophysiological properties of immunocytochemically identified rat dopamine neurons recorded in vitro. J Neurosci.

[56] Zhang J, Xu T-X, Hallett PJ, Watanabe M, Grant SGN, Isacson O (2009). PSD-95 uncouples dopamine-glutamate interaction in the D1/PSD-95/NMDA receptor complex. J Neurosci NIH Public Access.

[57] Zhang J, Saur T, Duke AN, Grant SGN, Platt DM, Rowlett JK (2014). Motor impairments, striatal degeneration, and altered dopamine-glutamate interplay in mice lacking PSD-95. J Neurogenet NIH Public Access.

[58] Pennuto M, Bonanomi D, Benfenati F, Valtorta F (2003). Synaptophysin I controls the targeting of VAMP2/ Synaptobrevin II to synaptic vesicles. Mol Biol Cell.

[59] Gordon SL (2014). Cousin M a. the sybtraps: control of synaptobrevin traffic by synaptophysin, α-synuclein and AP-180. Traffic.

[60] Shibaguchi H, Takemura K, Kan S, Kataoka Y, Kaibara M, Saito N (2000). Role of synaptophysin in exocytotic release of dopamine from Xenopus oocytes injected with rat brain mRNA. Cell Mol Neurobiol.

[61] Shin M-S, Jeong H-Y, An D-I, Lee H-Y, Sung Y-H (2016). Treadmill exercise facilitates synaptic plasticity on dopaminergic neurons and fibers in the mouse model with Parkinson’s disease. Neurosci Lett.

[62] Kamenetz F, Tomita T, Hsieh H, Seabrook G, Borchelt D, Iwatsubo T (2003). APP processing and synaptic function. Neuron.

[63] Calafate S, Buist A, Miskiewicz K, Vijayan V, Daneels G, de Strooper B (2015). Synaptic contacts enhance cell-to-cell tau pathology propagation. Cell Rep.

[64] Cirrito JR, Kang JE, Lee J, Stewart FR, Verges DK, Silverio LM (2008). Endocytosis is required for synaptic activity-dependent release of amyloid-β in vivo. Neuron.

[65] Wu JW, Hussaini SA, Bastille IM, Rodriguez GA, Mrejeru A, Rilett K et al (2016) Neuronal activity enhances tau propagation and tau pathology in vivo. Nat Neurosci Nature Publishing Group:1–11 10.1038/nn.4328 PMC4961585 27322420

[66] Imamura K, Sahara N, Kanaan NM, Tsukita K, Kondo T, Kutoku Y (2016). Calcium dysregulation contributes to neurodegeneration in FTLD patient iPSC-derived neurons. Sci Rep Nature Publishing Group.

[67] Yamamoto K, Tanei Z, Hashimoto T, Wakabayashi T, Okuno H, Naka Y (2015). Chronic Optogenetic activation augments Aβ pathology in a mouse model of Alzheimer disease. Cell Rep The Authors.

[68] Schultz MK, Gentzel R, Usenovic M, Gretzula C, Ware C, Parmentier-Batteur S (2018). Pharmacogenetic neuronal stimulation increases human tau pathology and trans-synaptic spread of tau to distal brain regions in mice. Neurobiol Dis Elsevier.

[69] Vazey EM, Aston-Jones G (2014). Designer receptors: therapeutic adjuncts to cell replacement therapy in Parkinson’s disease. J Clin Invest.

[70] Teresa M, Anno D, Caiazzo M, Leo D, Dvoretskova E, Medrihan L (2014). Remote control of induced dopaminergic neurons in parkinsonian rats. J Clin Invest.

[71] Aldrin-Kirk P, Heuer A, Wang G, Mattsson B, Lundblad M, Parmar M (2016). DREADD modulation of transplanted DA neurons reveals a novel Parkinsonian dyskinesia mechanism mediated by the serotonin 5-HT6 receptor. Neuron.

[72] Mcmahon T, Van Zijl PCM, Gilad AA (2015). L-type Calcium Channel blockers and Parkinson’s disease in Denmark. Ann Neurol.

[73] Chen Y, Xiong M, Dong Y, Haberman A, Cao J, Liu H (2016). Chemical Control of Grafted Human PSC-Derived Neurons in a Mouse Model of Parkinson’s Disease. Cell Stem Cell. Elsevier Inc.

[74] Pienaar IS, Gartside SE, Sharma P, De Paola V, Gretenkord S, Withers D et al (2015) Pharmacogenetic stimulation of cholinergic pedunculopontine neurons reverses motor deficits in a rat model of Parkinson ’ s disease. Mol Neurodegener Molecular Neurodegeneration:1–22 10.1186/s13024-015-0044-5 PMC4580350 26394842

[75] Stanojlovic M, Pallais Yllescas JP, Vijayakumar A, Kotz C (2019) Early sociability and social memory impairment in the A53T mouse model of Parkinson’s disease are ameliorated by Chemogenetic modulation of Orexin neuron activity. Mol Neurobiol Springer US:1–16 10.1007/s12035-019-01682-x PMC6842109 31250383

[76] Jiang L, Wu X, Wang S, Chen SH, Zhou H, Wilson B et al (2016) Clozapine metabolites protect dopaminergic neurons through inhibition of microglial NADPH oxidase. J Neuroinflammation 13 10.1186/s12974-016-0573-z PMC4869380 27184631

[77] Bærentzen S, Casado-Sainz A, Lange D, Shalgunov V, Tejada IM, Xiong M (2019). The Chemogenetic receptor ligand clozapine N-oxide induces in vivo Neuroreceptor occupancy and reduces striatal glutamate levels. Front Neurosci.

[78] Gomez JL, Bonaventura J, Lesniak W, Mathews WB, Sysa-Shah P, Rodriguez LA (2017). Chemogenetics revealed: DREADD occupancy and activation via converted clozapine. Science. American Association for the Advancement of Science.

[79] Jendryka M, Palchaudhuri M, Ursu D, van der Veen B, Liss B, Kätzel D et al (2019) Pharmacokinetic and pharmacodynamic actions of clozapine-N-oxide, clozapine, and compound 21 in DREADD-based chemogenetics in mice. Sci Rep 9 10.1038/s41598-019-41088-2 PMC6418145 30872749

