# Supplementary material for: Chronic nigral neuromodulation aggravates behavioral deficits and synaptic changes in an α-synuclein based rat model for Parkinson’s disease
Source: Acta Neuropathol Commun. 2019 Oct 22;7:160. doi: 10.1186/s40478-019-0814-3 (PMC6805517; doi:10.1186/s40478-019-0814-3)

Supplementary Figure 1

A.

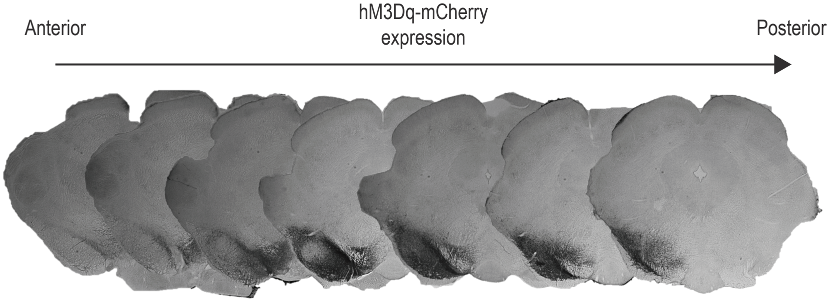

B.

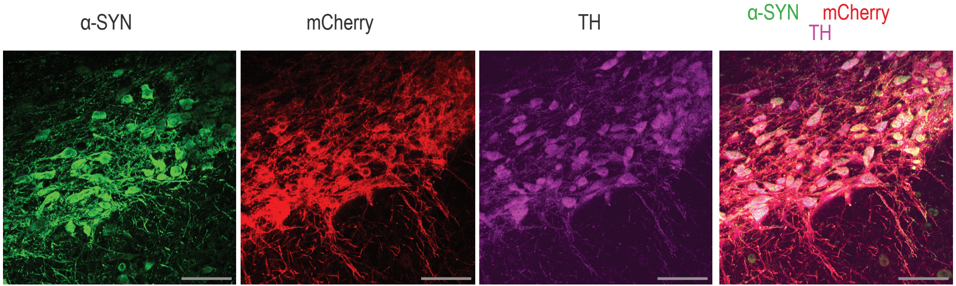

C.

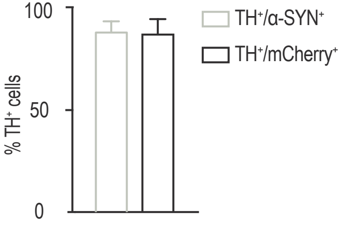

Supplementary Figure 2.

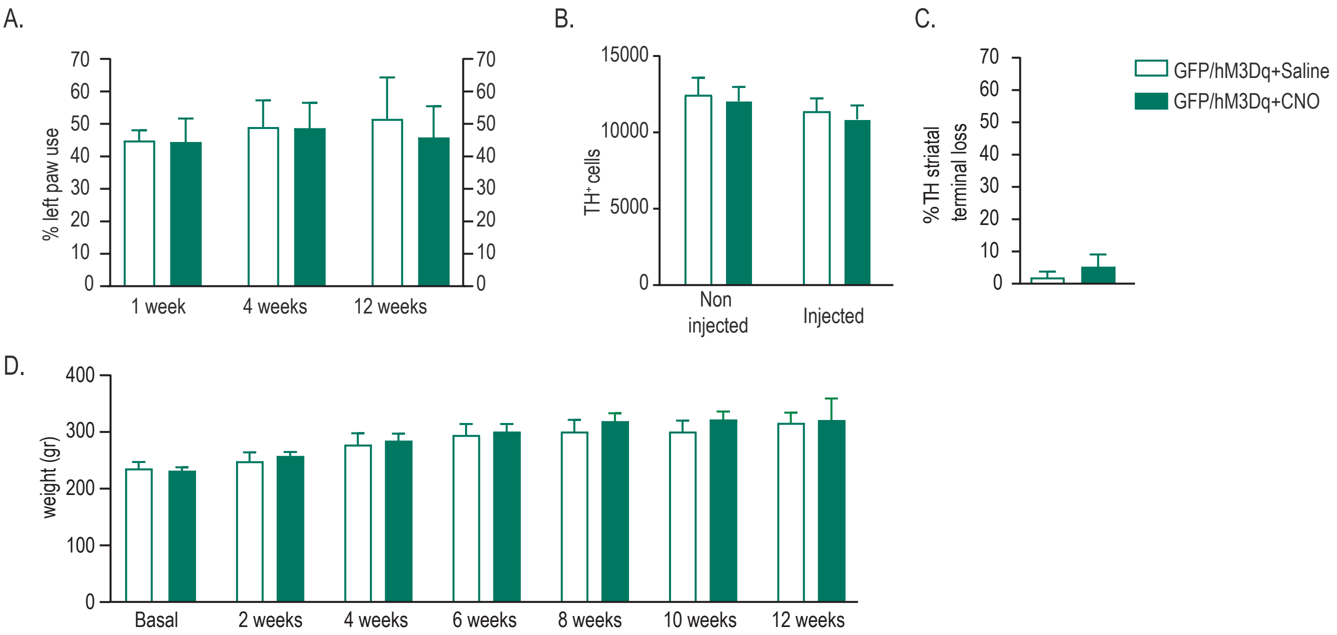

Supplementary Figure 3.

Uncut Western blot Figure 4. A

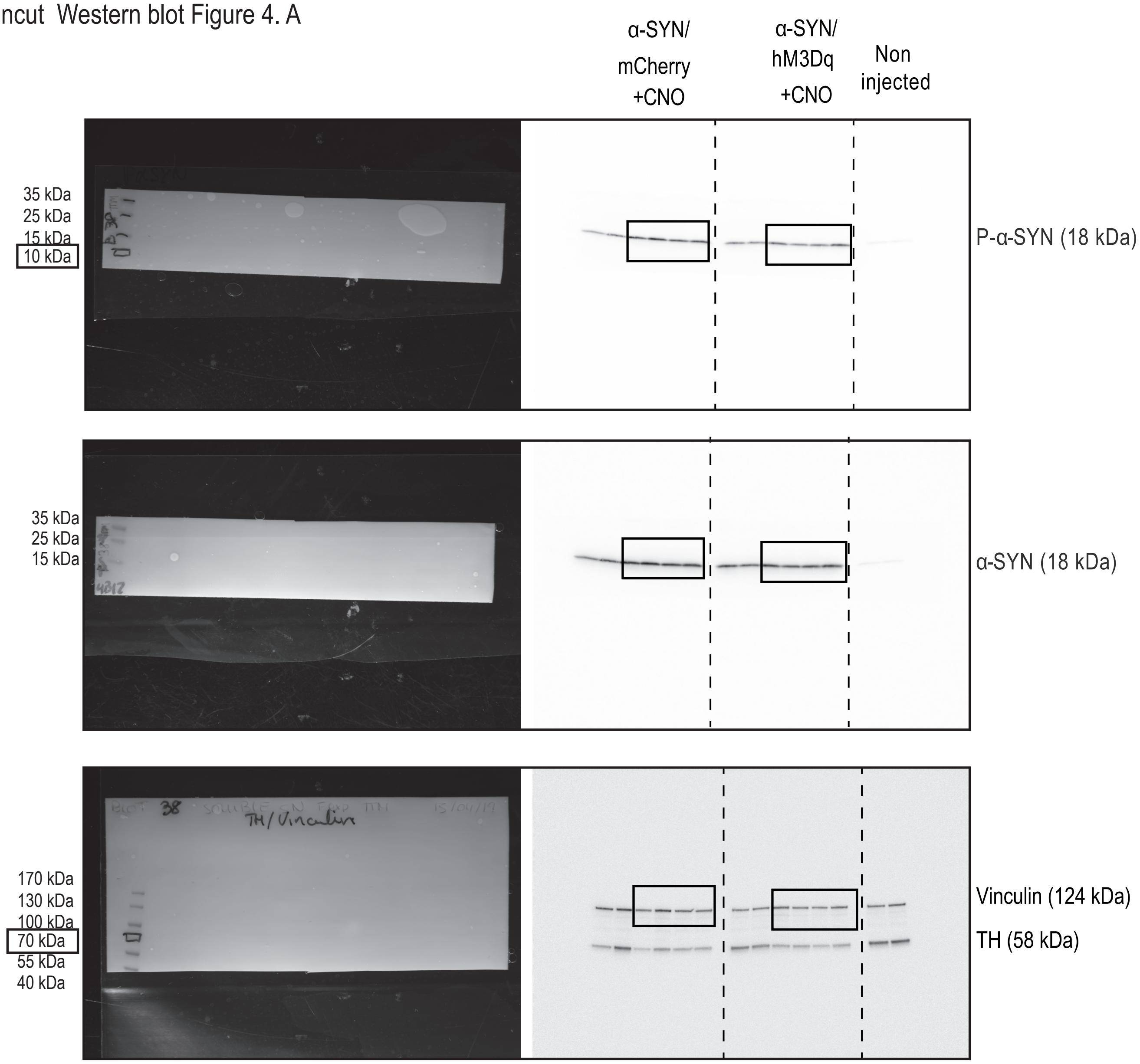

Uncut Western blot Figure 4. E

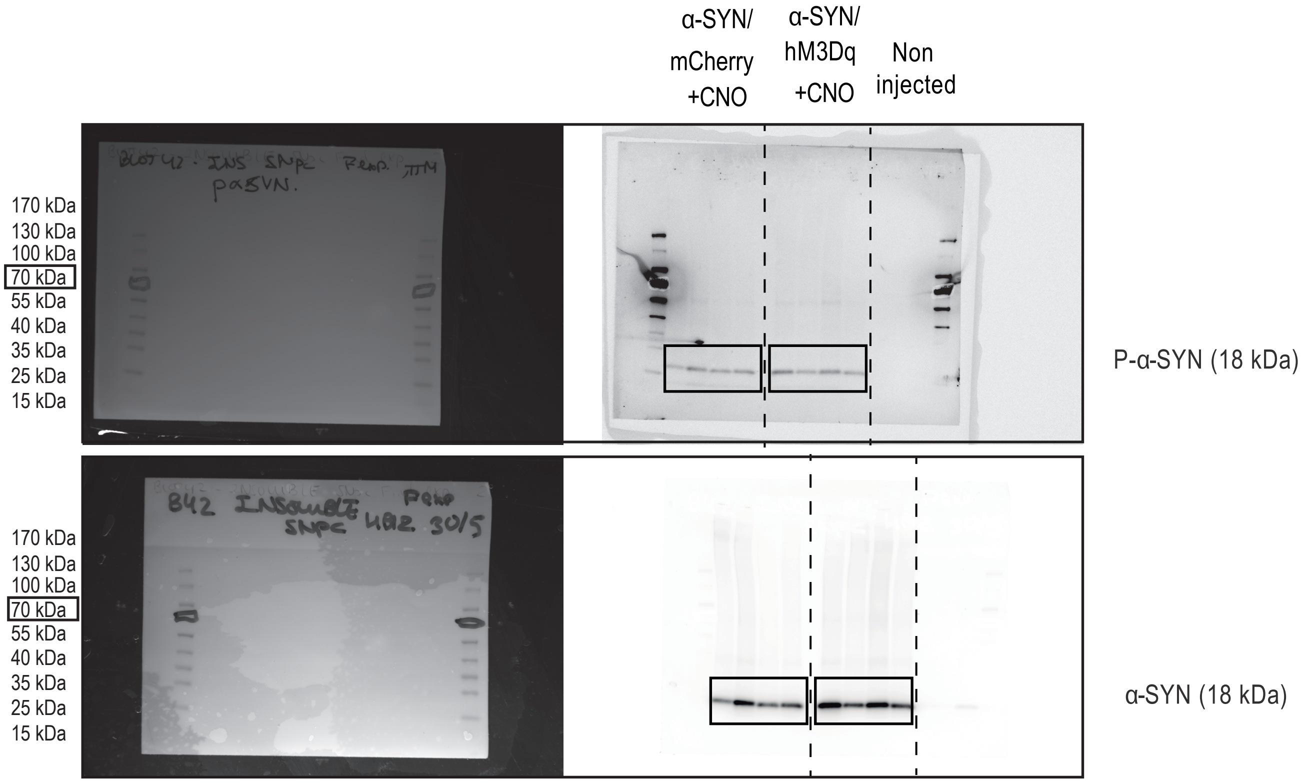

Uncut Western blot Figure 4. M

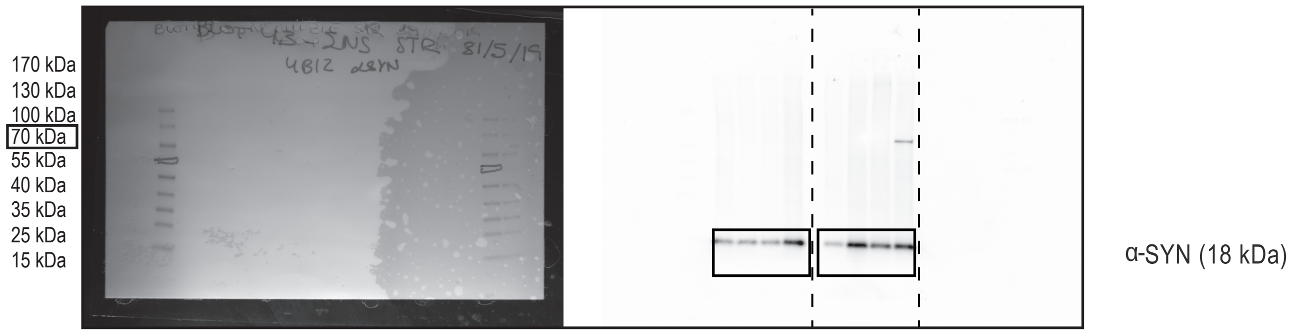

Uncut Western blot Figure 4. I

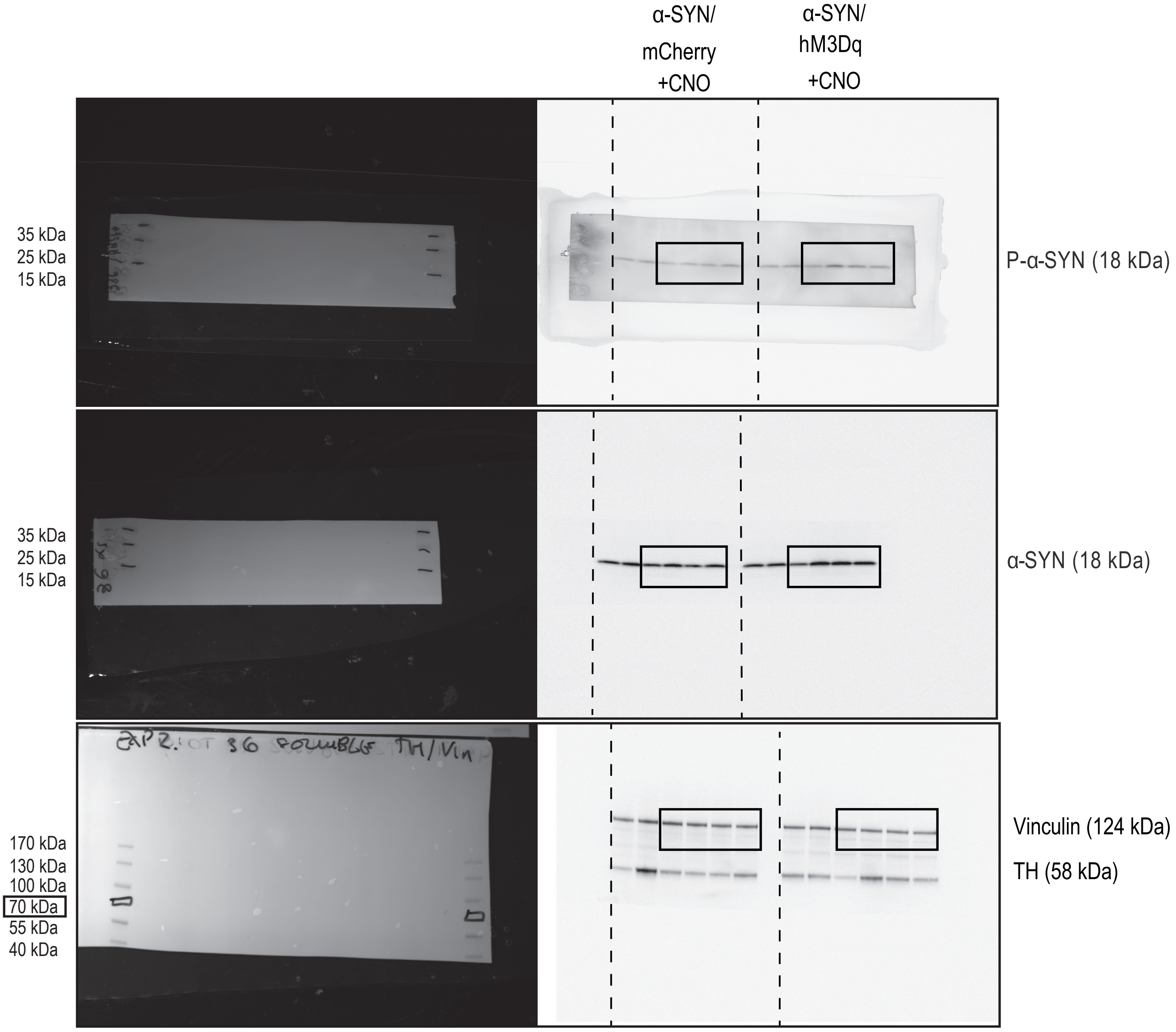

Supplementary Figure 4.

Uncut Western blot Figure 5. A

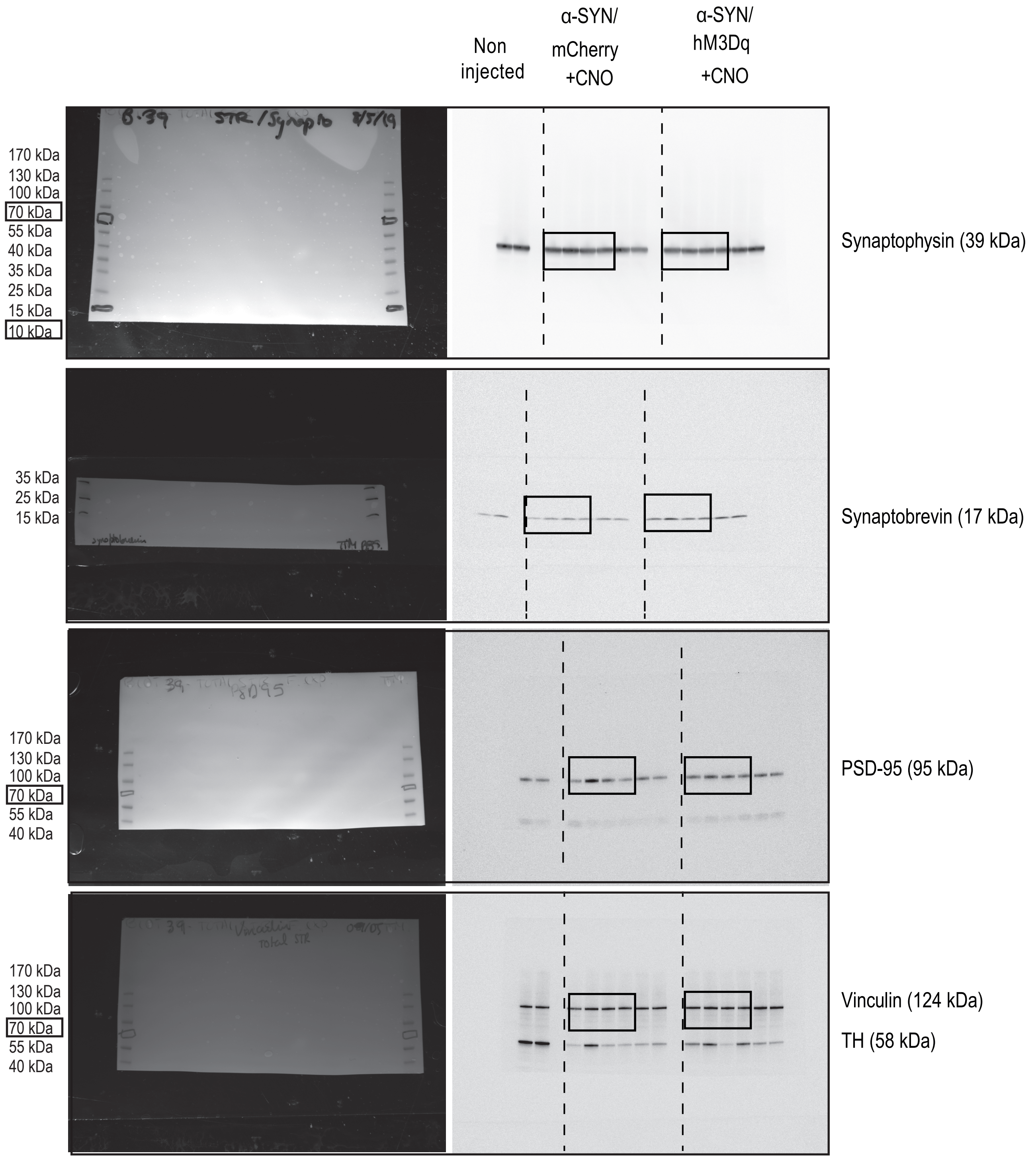

Supplement: Supplementary file 1 — Additional file 1: Figure S1. Transduction efficiency of rAAV2/7 α-SYN and rAAV2/8 hM3Dq vector (tagged with mCherry tag) in DN. Figure S2. Chronic neuronal activity modulation in rAAV2/7 GFP vector injected animals does not affect motor behavior or TH+ cell numbers. Figure S3. Complete and uncropped western blots from Fig. 4. Figure S4. Complete and uncropped western blots from Fig. 5. [file 40478_2019_814_MOESM1_ESM.pdf]
